# Multidisciplinary diagnostic and therapeutic approach to acute mesenteric ischaemia: A case report with literature review

**DOI:** 10.1177/2050313X211004804

**Published:** 2021-05-20

**Authors:** Jurij Janež, Jasna Klen

**Affiliations:** 1 Department of Abdominal Surgery, University Medical Centre Ljubljana, Ljubljana, Slovenia; 2 Faculty of Medicine, University of Ljubljana, Ljubljana, Slovenia

**Keywords:** Acute mesenteric ischaemia, arterial embolism, aetiology, diagnostic approach

## Abstract

Superior mesenteric artery embolisation is the most common cause of acute mesenteric ischaemia. Superior mesenteric artery embolisation can be caused by various cardiac diseases (myocardial ischaemia or infarction, atrial tachyarrhythmias, endocarditis, cardiomyopathies, ventricular aneurysms and valvular disorders), arterial aneurysms, ulcerated atherosclerotic plaques of the major arteries and others. A case of 65-year-old, previously healthy man with superior mesenteric artery embolism, who was found to also have mural aortic thrombi, is presented. The patient underwent an emergency procedure; small intestine and cecum were resected and jejuno-ascendo anastomosis was performed. The patient was put on lifelong anticoagulation therapy. Neither cardiac diseases nor arterial aneurysms were detected. There were no signs of underlying atherosclerosis. Work-up for antiphospholipid antibodies and rheumatic diseases was negative. Tumour markers were within normal levels and blood cultures were negative. This case represents the challenges in recognising an underlying cause of acute mesenteric embolism and highlights the importance of multidisciplinary diagnostic and treatment approach.

## Introduction

Acute mesenteric ischaemia (AMI) is a surgical emergency that occurs due to impairment of mesenteric arterial or venous circulation, with a reported incidence rate of 12.9 per 100,000 person-years and with high mortality rate (50%–70%).^1^ There is inadequate blood supply, which leads to ischaemia and inflammatory injury. A consequence of both is necrosis of the bowel wall. AMI can be non-occlusive or occlusive. AMI is caused by superior mesenteric artery (SMA) embolisation (40%–50%), followed by SMA thrombosis (15%–25%), mesenteric venous thrombosis (5%–15%) and non-thrombotic mesenteric ischaemia (20%).^2–4^

In 2017, the World Society of Emergency Surgery provided guidelines for evaluation and potential treatment of suspected AMI. The recommendations were based on the most currently accepted concepts in the management of AMI. Although various potential aetiologies of AMI are clearly stated, the postoperative management has unfortunately not yet been elucidated, despite the fact that numerous studies have already been performed.^4^

Herein, a case of 65-year-old, previously healthy man with SMA embolism, who was found to also have mural aortic thrombi, is presented. The purpose of this report is to systematically review potential aetiologies of acute mesenteric embolism and to highlight the importance of the multidisciplinary diagnostic and treatment approach.

## Case report

**Figure 1.**
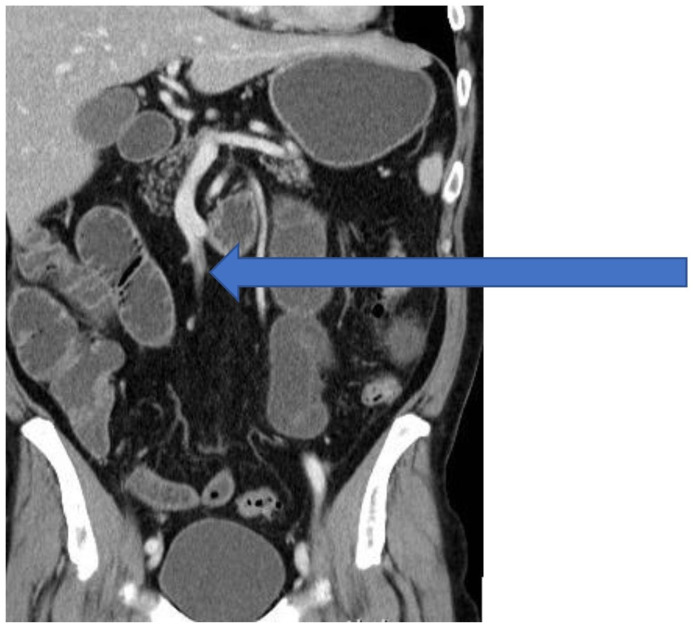
Preoperative angiographic computed tomography showed an acute completely embolic occlusion of at least three out of five branches of arteria mesenterica superior (arrow).

**Figure 2.**
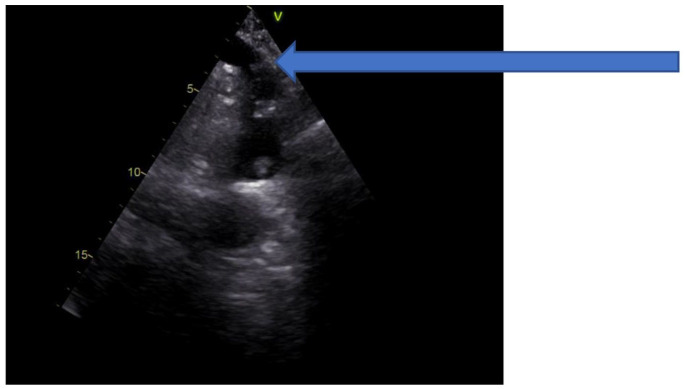
Transthoracic echocardiogram presenting unclear floating structure in ascending aorta (arrow).

A 65-year-old Caucasian non-smoker male was admitted to our emergency department due to a 2-day history of abdominal pain, nausea and diarrhoea. He had no records of any prior illnesses or surgeries. There was no relevant family history. The patient denied alcohol or drug abuse. On admission to the emergency department, he was mildly tachycardic while the blood pressure was in normal ranges. Physical examination was positive for diffuse abdomen tenderness. Digital rectal examination was normal. Laboratory tests showed elevated serum lactic acid (2.70 mmol/L) and C-reactive protein (350 mg/L), leukocytosis (21.8 × 10^9^/UL) and normal levels of the coagulation parameters (prothrombin time (PT) 0.70; PT international normalised ratio (PT-INR) 1.23). The serum levels of cholesterol (4.8 mmol/L), fibrinogen (2.8 g/L), thrombocytes (210 × 10^9^/L), erythrocytes (5.2 × 10^12^/L) and haemoglobin (152 g/L) were within normal range. The plain abdominal X-ray demonstrated only dilated loops of small bowel measuring 5 cm. He underwent an abdominal computed tomography (CT) scan with intravenous contrast, demonstrating an acute totally embolic occlusion of at least three out of five branches of arteria mesenterica superior (AMS) and as a result, the signs of ischaemia and ileus of small bowel and pneumatosis, as shown in Figure 1. Small infarctions of the spleen were also visible. Interventional radiologist and cardiovascular surgeon were consulted and neither interventional nor cardiovascular therapy was indicated. Patient underwent an emergency procedure under general anaesthesia. Due to an ischaemic and necrotic tract of small bowel, small intestine and cecum were resected and jejuno-ascendo anastomosis was performed to re-establish the bowel continuity. There remained only 1 m of small bowel and almost entire colon. Temporary abdominal closure with an abdominal vacuum-assisted closure (VAC) dressing was applied. Scheduled second look was given after 48 h, to find a completely viable remaining bowel. Pathology examination revealed an ischaemic enteritis with focal transmural haemorrhagic infarction involving ileum. The patient stayed in the intensive care unit (ICU) for 6 days. He was therapeutically anticoagulated with continuous intravenous standard heparin infusion. Electrocardiogram (ECG) and troponin were normal. Transthoracic echocardiogram was performed, showing a heart of normal dimensions and contractility, as well as normal valve function without vegetations and no tumour masses (myxoma), as shown in Figure 2. On the contrary, a friable, mobile mass measuring 1.1 cm × 2.3 cm, arising from the wall of the aortic arch, was found. The mass was confirmed by trans-oesophageal echocardiography (TEE). A CT angiography (CTA) of the heart and thoracic aorta was obtained to further define the extent and nature of the lesion, confirming two mural aortic thrombi just before the division of brachiocephalic trunk. A screening for antiphospholipid antibodies (anti-cardiolipin antibodies (aCL), anti-β_2_-glycoprotein 1 antibodies (anti-β2GP1), lupus anticoagulant) and rheumatic diseases (HEp-2 test, anti-ENA, ANCA) were negative. Postoperatively, the abdominal CT scan showed occlusion of AMS, connections of ileal arteries with the right colic artery, forming arterial arcade and largest area of splenic infarction. Patient was discussed at the multidisciplinary cardiovascular meeting, which decided that the patient will be treated with low molecular weight heparin, without several other treatment modalities, such as thrombolysis, thromboendarterectomy or surgical resection with graft interposition. The patient was put on lifelong anticoagulation therapy with warfarin. Due to a 6-month history of dry, non-productive cough, he underwent the chest X-ray, which did not show any abnormalities. There were no other respiratory symptoms. Later on, the patient stopped coughing, so further diagnostic, such as chest CT, was not performed. During the follow-up, a cardiac ultrasound was performed and no aortic thrombi were visible.

## Discussion

The SMA normally serves as the primary arterial supply of the jejunum, ileum and colon to the level of the splenic flexure. SMA is the primary blood supply for the small bowel with some collateral flow from the celiac arterial system, via the superior and inferior pancreaticoduodenal arteries, as well as from the inferior mesenteric artery. Intestinal blood returns via the portal vein. The splanchnic circulation receives 15%–35% of the cardiac output, depending on the feeding state, but oxygen extraction is relatively low, accounting for the oxygen delivery capacity of the portal vein to the liver. Thus, blood supply must be reduced by more than 50% before the small intestine becomes ischaemic.^5^ The severity of ischaemia depends on the affected vessel, the extent of collateral vessel blood flow and the time of duration. Thrombotic occlusions are often located more proximal within the SMA and are associated with a more extensive intestinal infarction than embolic occlusions.^6^ An acute mesenteric arterial embolism usually spares the proximal jejunum and colon and it is in more than 20% of cases associated with concurrent emboli, classically with embolisms to spleen or kidney.^4^ In case of our patient, embolic occlusion of SMA was found. At least three out of five branches of AMS were completely occluded. Regarding the histology report, ischaemic enterocolitis with segmental transmural gangrene, most of the ileum was present, while resection edges were not impaired. Concurrent emboli to the spleen causing spleen infarcts were also observed.

Optimal treatment of AMI may include both revascularisation and resection of necrotic gut.^7–10^ Due to clinical and radiologic signs of advanced intestinal ischaemia (peritoneal irritation and CT signs of intestinal wall pneumatosis and air in portal venous system), immediate laparotomy was indicated.^7,9^ Ischaemic bowel (jejunum, ileum and cecum) was resected. At that point, some controversies regarding the appropriate timing of anastomosis formation could be found. Some authors suggest formation of anastomosis during the first surgical procedure with a planned second-look surgery in normotensive patient without intra-abdominal sepsis or peritonitis, if there is no doubt about the viability of the remaining bowel.^11,12^ However, the others propose delayed bowel reconstruction until the second or third look due to high risk of anastomotic leakage.^10^ In our patient, jejuno-ascendo anastomosis was performed because the remaining small bowel and colon were completely viable, a temporary abdominal closure with an abdominal VAC dressing was applied and second-look procedure was performed within 48 h, showing viable remaining bowel. However, SMA embolectomy was not performed due to a 2-day history of abdominal pain, nausea, diarrhoea and CT signs of advanced AMI.

AMI often affects elderly patients with ischaemic heart disease and atrial fibrillation. When this disease occurs in young or middle-aged otherwise healthy adults, recognising an underlying cause may be a challenging and demanding task.^6^

As most mesenteric emboli originate from a cardiac source, common risk factors are myocardial ischaemia or infarction, atrial tachyarrhythmias, endocarditis, cardiomyopathies, ventricular aneurysms and valvular disorders. Occasionally, emboli are generated from an atherosclerotic aorta. Emboli typically lodge at points of normal anatomic narrowing, and the SMA is particularly vulnerable because of its relatively large diameter and low takeoff angle from the aorta. The majority of emboli lodge 3–10 cm distal to the origin of the SMA, thus classically sparing the proximal jejunum and colon.^13^ In the presented case, no arrythmias were detected with the ECG test and troponin levels were within normal levels. Two-dimensional echocardiography, accompanied by TEE and CTA was an essential examination, which revealed two mural aortic thrombi arising just before the division of brachiocephalic trunk without any signs of infection. Neither structural nor contractility cardiac abnormalities were found. In the absence of structural heart defects, a patent foramen ovale and, consequently, paradoxical embolism were also excluded.

Arterial aneurysms followed by ulcerated atherosclerotic plaques of the major arteries are the second most common source of the emboli.^14^ Unlike our patient, most patients with aortic embolic disease secondarily to atherosclerosis, are typically elderly with multiple comorbid conditions.^15^ In our otherwise healthy patient, lipid profile was within normal levels, and by imaging tests, neither localised nor generalised atherosclerotic disease was detected. Consequently, antiplatelet drugs and statin were not initiated.

While the aortic thrombus in the setting of underlying atherosclerosis is common, the association of aortic thrombus with a hereditary thrombophilia is relatively rare.^15^ Hereditary thrombophilia, an enhanced inherited tendency to form intravascular thrombi, is associated with deficiencies of protein C, protein S, antithrombin and activated protein C resistance (factor V Leiden mutation).^16^ Reported a 48-year-old woman, who was diagnosed with thromboembolism of the mesentery artery and multiple mural thrombi of the thoracic aorta, associated with combined protein C and S deficiency. In addition, the case reported by Baburaj et al.^17^ describes a patient in the fourth decade of life with thrombosis in AMS, in which haematological work-up detected a protein S deficiency. Every patient with a spontaneous arterial thrombosis and without any known risk factors should be screened for thrombophilia. Unfortunately, screening of inherited thrombophilia was not performed in our patient, due to the prior introduction of heparin therapy. Therefore, suspecting hereditary thrombophilia, as an underlying cause of embolism to AMS at the time of admission, may lead to timely haematological work-up, as obviously seen from the previously mentioned case reports.^15,17^ However, knowing that patient has inherited thrombophilia has no impact on the therapeutic approach. Patients with hereditary thrombophilia, as well as patients whose reasons for thromboembolic state are unknown, tend to be on lifelong anticoagulation therapy, as they are more susceptible for recurrent unprovoked, life-threatening thromboembolic event. While guidelines for optimal treatment of venous thrombotic or embolic events are well-defined, those for arterial thromboembolism are still not available.^18,19^

Occurrence of arterial thromboembolic event may indicate testing for acquired thrombophilia–antiphospholipid syndrome, as well as underlying connective tissue diseases or rheumatologic conditions (systemic lupus erythematosus (SLE), vasculitis).^4^ The results of testing for antiphospholipid antibodies and vasculitis were negative. One could speculate that aortic thrombus might have developed due to infectious aortitis. Most commonly, it is associated with *Salmonella* and *Staphylococcal* species alongside with *Streptococcus pneumonia*, while tuberculous, syphilitic or luetic aortitis occurs rarely in the developed world.^20^ However, our patient showed negative blood culture results and magnetic resonance imaging (MRI) of thoracic aorta exhibited no signs of vasculitis. Therefore, aortitis-mediated mechanism is unlikely to explain the presented observation. Among rare causes of infective aortitis could be an infection with SARS-CoV-2 virus, which is known to have prothrombotic tendencies and can cause thrombosis of unusual sites (ischaemic limb, mesenteric artery thrombosis, aortic thrombosis).^21,22^ Infection with SARS-Cov-2 virus could be one of possible causes of aortic thrombosis and SMA embolism in our patient. However, our patient was not tested for SARS-CoV-2 infection, because this case was managed before SARS-CoV-2 pandemics.

Correlations with rare sites of venous or arterial thrombosis and many myeloproliferative disorders or clonal disorders with acquired bone marrow, such as paroxysmal nocturnal haemoglobinuria (PHN) are also well known.^23^ Again, the results of blood and urine examination indicated no underlying haemolysis.

The coexistence of embolic occlusion (of SMA) and arterial thrombosis may indicate a severe prothrombotic state, such as a paraneoplastic syndrome with prothrombotic tendencies, most commonly associated with lung, gastric and pancreatic malignancies.^24,25^ Hypercoagulability in paraneoplastic syndrome occurs due to a complex interaction among cancer cells, host cells and the coagulation system.^24^,^26^ However, deep-vein thrombosis and pulmonary embolism are more commonly observed than arterial thrombosis.^27^ Age-appropriate cancer screening is recommended, but extensive malignancy work-up in the absence of any clinical factors, suggesting underlying malignancy, is discouraged.^25,28^ In our patient, coexistence of prothrombotic state and history of 6-month cough may lead to a suspicion of the first manifestation of unrecognised lung carcinoma. Chest X-ray was performed and no masses were found, which do not exclude lung carcinoma. Sometimes paraneoplastic syndromes manifest 6–15 months before occult cancer could be detected.^29,30^ However, our patient stopped coughing later and was fully asymptomatic regarding the lung disease, so further diagnostic, such as chest CT was not performed. The tumour markers for visceral cancers were negative, but for lung cancer, at the moment there is no valuable tumour marker. The chest x-ray was also normal. The abdominal CT after surgery was performed and it did not show any suspicious changes, suggesting abdominal malignancy.

The World Society of Emergency Surgery provided guidelines for evaluation and potential treatment of suspected AMI. The recommendations were based on the most currently accepted concepts in the management of AMI. Various potential aetiologies of AMI are clearly stated, the postoperative management has unfortunately not yet been elucidated.^4^ According to our experiences, the treatment of AMI with confirmed bowel ischaemia and gangrene is primarily surgical with necrotic bowel resection and performing an anastomosis or a stoma, if the remaining small bowel length is satisfactory for surviving. The multidisciplinary approach has to be attained for elucidating a cause of AMI, which can be multifactorial. Paraneoplastic syndrome and an undelaying cancer must be suspected, as also other diseases, which lead to prothrombotic and hypercoagulability state. But sometimes, the AMI can be idiopathic when the exact cause, despite the extended diagnostic, cannot be confirmed.

## Conclusion

In summary, the case of 65-year-old previously healthy man with SMA embolism, who was found to also have mural aortic thrombi, is presented. On the basis of our findings, the exact aetiology behind the embolic occlusion of the SMA is uncertain. Considering lack of cardiac sources and hypercoagulable conditions, fragmentation of the aortic mural thrombi may most likely be the cause of SMA embolism. Manifestation of paraneoplastic syndrome prior to detection of underlying malignancy could also be the underlying cause, but was not confirmed during the patient follow-up. Hereditary thrombophilia should be included in the differential diagnosis, although screening of inherited thrombophilia was not performed due to the prior introduction of low molecular weight heparin therapy. The possible cause of aortic thrombosis could be an infection with SARS-CoV-2 virus. Even though, as AMI usually occurs in elderly patients with ischaemic heart disease and atrial fibrillation, younger patients with AMI should also be evaluated carefully. Multidisciplinary diagnostic and treatment approach should be used as it may improve prognosis and survival rate. However, despite extended diagnostics, many cases of AMI remain without knowing the exact cause.
